# ABL1 in thalamus is associated with safety but not fear learning

**DOI:** 10.3389/fnsys.2013.00005

**Published:** 2013-03-26

**Authors:** Mouna R. Habib, Dan A. Ganea, Ira K. Katz, Raphael Lamprecht

**Affiliations:** Sagol Department of Neurobiology, Faculty of Natural Sciences, Center for Gene Manipulation in the Brain, Center for Brain and Behavior, University of HaifaHaifa, Israel

**Keywords:** safety learning, fear learning, thalamus, ABL1, memory

## Abstract

In auditory fear conditioning a tone is paired with a footshock, establishing long lasting fear memory to the tone. In safety learning these stimuli are presented in an unpaired non-overlapping manner and enduring memories to the tone as a safety signal are formed. Although these paradigms utilize the same sensory stimuli different memories are formed leading to distinct behavioral outcome. In this study we aimed to explore whether fear conditioning and safety learning lead to different molecular changes in thalamic area that receives tone and shock inputs. Toward that end, we used antibody microarrays to detect changes in proteins levels in this brain region. The levels of ABL1, Bog, IL1B, and Tau proteins in thalamus were found to be lower in the group trained for safety learning compared to the fear conditioning group 6 h after training. The levels of these proteins were not different between safety learning and fear conditioning trained groups in auditory cortex. Western blot analysis revealed that the ABL1 protein level in thalamus is reduced specifically by safety learning but not fear conditioning when compared to naïve rats. These results show that safety learning leads to activation of auditory thalamus differently from fear conditioning and to a decrease in the level of ABL1 protein in this brain region. Reduction in ABL1 level in thalamus may affect neuronal processes, such as morphogenesis and synaptic efficacy shown to be intimately regulated by changes in this kinase level.

## Introduction

Fear conditioning leads to long-term fear memory formation and is a model of psychopathologies conditions such as anxiety and post-traumatic stress disorders (e.g., LeDoux, 2000). On the other hand, in safety learning the subject learns the safety properties of a signal—a useful relief from a fearful situation and chronic stress (e.g., Rescorla, 1969). Although fear and safety learning can be formed by the same sensory stimuli different memories are created. We are therefore interested to unveil whether fear and safety learning paradigms activate different cellular processes during memory formation. Toward that end we aimed to identify molecular events initiated by fear conditioning or safety learning.

In fear conditioning a tone (conditioned stimulus; CS), is paired with a mild footshock (unconditioned stimulus; US) leading to long lasting fear memories, such that on subsequent occasions the CS comes to elicit behavioral, autonomic, and endocrine responses that are characteristically expressed in the presence of danger (Fanselow and LeDoux, 1999; LeDoux, 2000; Davis and Whalen, 2001; Sah et al., 2003; Maren, 2005). In safety learning the tone and shock are presented in an unpaired non overlapping manner and the tone is memorized as a safety signal that predicts the absence of the shock (Rogan et al., 2005; Pollak et al., 2008; Ostroff et al., 2010). Fear conditioning and safety learning elicit different cellular and molecular responses in lateral amygdala (LA) and Caudoputamen (CP) (Rogan et al., 2005; Pollak et al., 2008). Here we explored whether fear conditioning and safety learning lead to different molecular responses in the auditory thalamus which transfers information to both LA and CP during fear and safety learning (LeDoux, 2000; Rogan et al., 2005). Lesion to the auditory thalamus was shown to impair fear conditioning memory formation (LeDoux et al., 1986). Furthermore, fear conditioning induces frequency-specific receptive field plasticity in the medial geniculate body (Lennartz and Weinberger, 1992). In contrast, evidence show that the auditory thalamus is not involved in contextual fear conditioning. For example, pre-training intra-MGm (medial division of the medial geniculate body) thalamic infusion of the NMDA receptor antagonist (APV), which attenuates synaptic transmission in the thalamus, impaired the acquisition of auditory but not contextual fear conditioning (Webber et al., 1999; Maren et al., 2003). The auditory thalamus is also needed for safety learning. Lesion to the auditory thalamus post training impaired the ability to inhibit fear in the presence of the noise safety signal (Heldt and Falls, 2006) and changes in CS response after safety learning in LA and CP is consistent with modulation of CS information arriving via a direct thalamic projection from MGm/PIN (posterior intralaminar nucleus) (Rogan et al., 2005). Studies have shown that both tone and footshock arrive to the MGm, PIN, and SG (suprageniculate nucleus) area of the thalamus (Bordi and LeDoux, 1994). Convergence of auditory and footshock responses was also detected in these areas.

The aforementioned studies show that the auditory thalamus is needed for both fear conditioning and safety learning. The thalamus might serve as a passive sensory station not affected by different temporal activation of its neurons or be activated differently by fear and safety learning leading to alteration in cellular responses needed for the establishment of different memories. To explore this possibility we aimed in this study to identify changes in specific proteins levels in thalamus after fear conditioning and safety learning. Ample studies show a role for protein synthesis and degradation in synaptic plasticity and memory formation following different types of behavioral paradigms mediated by different brain regions (Davis and Squire, 1984; Steward and Schuman, 2003; Fioravante and Byrne, 2011; Gal-Ben-Ari et al., 2012). In auditory thalamus protein synthesis is needed for fear conditioning memory formation as injection of the protein synthesis inhibitor anisomycin into the thalamus 30 min before fear conditioning impaired long-term fear memory formation (Parsons et al., 2006). Moreover, intra MGm/PIN infusion of antisense ODN against EGR-1 90 min prior to fear conditioning impaired fear LTM (Overeem et al., 2010). Increasing CREB in MGm and PIN enhanced formation of an auditory conditioned fear memory (Han et al., 2008).

We utilized the antibody microarray and Western blot approaches to identify possible changes in proteins levels in thalamus between fear conditioning and safety learning.

## Materials and methods

### Animals

Male Sprague Dawley rats (200–224 g) were used (Harlan Laboratories Jerusalem). Animals were housed individually in clear plastic cages and maintained at 22 ± 2°C in a 12 h light/dark cycle, with free access to food and water. Behavioral experiments were approved by the University of Haifa Institutional Committee for animal experiments in accordance with National Institutes of Health guidelines.

### Behavioral procedures

Fear conditioning took place in a Plexiglas rodent conditioning chamber with a metal grid floor dimly illuminated by a single house light and enclosed within a sound attenuating chamber. Rats were habituated to the training chamber for 3 days (17 min/day) before training. Rats for the summation test were not habituated. The next day the rats were trained for fear conditioning and presented with five pairings of a tone (CS; 20 s, 5 kHz, 75 dB) that co-terminated with a footshock (US; 0.5 s, 1.3 mA). The inter-trial interval is random with average of 120 s. Safety learning took place in the same conditioning chamber. Rats received non-overlapping five presentations of the CS and US where the US preceded the CS by 60 s and at least 120 s was required between a tone CS and the next trial. Naïve group were introduced to the training cage with no CS or US. For the retardation test, rats were given two tone shock pairings (0.4 mA, 1-s shock, ITI = 180 s) one day after safety learning training.

Groups were tested in a different chamber with dark Plexiglas walls and Formica floor. In the summation test rats were tested in the same context where they were conditioned. Fear and safety responses were quantified by measuring the amount of time spent freezing during five CS presentations (20 s, 5 kHz, 75 dB) with average ITI of 180 s. The video images of rats during testing were transferred to a computer (Dell OptiPlex GXpro) equipped with an analysis program (Image) and a macroprogram (P. Schmid, Behavioral Neurobiology Laboratory, Swiss Federal Institute of Technology Zurich). The percentage of changed pixels between two adjacent 1 s images was calculated and if the percentage of change in images was <0.05%, the behavior of the rat was scored as “freezing” for the respective later second. In microarray experiments we tested a group of animals for fear conditioning or safety memory 24 h after training to verify that fear or safety learning occurred. This group was trained with the other animals that their tissue was processed for microarray. For Western blots analysis we tested all rats 6 h after training. Animals with percent freezing criterion of above 60% for Paired, below 45% for Unpaired and below 10% for Naïve groups were further sacrificed for Western blot analysis. Animals that did not reach criteria were removed from the experiments.

### Tissue dissection

Six hours after training the brains were quickly frozen on dry ice and kept at −80°C until use. The brains were sliced at the thickness of 40 μm using Leica CM1900 cryostat until the thalamic area containing the PIL/MGm, was visualized. The brain area was micropunched from frozen brains with blunted 2 mm diameter sample corer (Fine science tools, CA, USA). For the microarray: tissue from fear conditioning trained animals (*n* = 11) or from safety learning trained group (*n* = 11) was combined to reach the protein level needed for the assay. For Western blots analysis: two thalamic regions from each rat were combined for analysis. Few slides were collected on Super/Plus Microscope Slides (Fisher Scientific, USA) for histology of dissection. All samples were stored at −80°C until further use.

### Histology

Brain slices were stained with methylene blue and punched areas were verified using Olympus IX81 microscope (×1.25 magnification). Brains with incorrect dissection areas were removed from the experiments.

### Antibody microarray

To identify changes in proteins level in thalamus between fear conditioning and safety learning we utilized the Clontech antibody array that consists over 500 individual antibodies spotted in duplicates. The protocol used was as recommended by the manufacturer. The brain tissue was rapidly transferred to a prechilled mortar containing Alumina, and immediately homogenized in iced cold homogenization buffer (Extraction/Labeling Buffer). Homogenate was centrifuged for 30 min at 10,000 g. Protein concentration was measured using Pierce's BCA Protein assay Reagent Kit. Cy3 and Cy5 were dissolved in 110 μl of Extraction/Labeling Buffer. The homogenate supernatants and Cy3 and Cy5 were mixed in four tubes as follows: A-Paired supernatant and Cy5; B-Unpaired supernatant and Cy3; C-Paired supernatant and Cy3; D-Unpaired supernatant and Cy5. The four tubes were incubated covered with foil for 90 min on ice. Four microliters of blocking buffer was added followed by incubation for 30 min on ice. Unbound dye was removed by PD-10 desalting columns equilibrated with 3 × 5 ml of 1× Desalting Buffer. Protein samples were eluted and protein concentration was measured using Pierce's BCA. One hundred μg of proteins from samples were mixed as follows: Tubes A and B to mix 1 and tubes C and D to mix 2. Twenty μg of mix 1 was added to microarray 1 and 20 μg of mix 2 to microarray 2. Each slide was incubated with the samples in incubation chamber for 40 min at room temperature (RT), followed by washing procedure as described in details in Clontech protocol.

### Data analysis

Data analysis was done as instructed by supplier. The slides were scanned using Axon GenePix 4000B scanner. The sequence text files were analyzed with Clontech software to produce the scatter plots and correlation values. Subsequently the scanner files were analyzed with an AB Microarray Analyzing Workbook supplied by manufacturer to calculate internally normalized ratios (INR) using the conversion of fluorescence data to INRs for each coordinate in the array. The replicated values within each slide were averaged and INR was calculated as follows INR = √Ratio1/Ratio2 where ratios 1 and 2 correspond to slide 1 and 2. Ratio 1 = Paired-Cy5/Unpaired-Cy3. Ratio 2 = Unpaired-Cy5/Paired-Cy3. The average INR is calculated for each antibody. Values that are ≥1.3 or ≤0.77 × averaged INR indicate valid changes that signify differences in protein abundance.

### Western blot

Thalamus tissue was homogenized, in glass homogenizer using Teflon pestle in 300 μl homogenization buffer [(in mM: HEPES 10, EDTA 2, EGTA 2, DTT 0.5, 1% phosphatase inhibitor cocktail (Sigma), and 1% protease inhibitor cocktail (Sigma)].

After centrifugation at 10,000 g for 5 min at 4°C, 50 μl of lysates were kept for protein quantification and 200 μl of lysates were transferred to 1.5 ml eppendorf tubes containing 2× SDS-sample buffer, boiled for 5 min and stored at −80°C. Protein content of lysates was determined using the Bradford protein assay (Biorad). Proteins (total 10 μg for each sample) were separated by 7.5% SDS-PAGE and transferred to PVDF membrane (Millipore, immobilon-p 0.2 μm). Blots were incubated in blocking buffer [(in 5% non-fat dry milk or 3% BSA (depending on primary antibodies as recommended) in Tris buffered saline containing 1% Tween-20 (TBST)] for 1 h at RT, washed 3 × 10 min in TBST and incubated with primary antibodies to detect ABL1 (1:3000; over night in 4°C; BD Biosciences 554148), Bog (1:1000; over night in 4°C; Transduction laboratories B12520), Tau (over night in 4°C; BD Biosciences; 556319) or β-Tubulin (1:30000 for 1 h at RT; Sigma, T2200) in blocking buffer on rotating mixer. The blots were washed twice with TBST and incubated with either anti-mouse (1:2000 in 5% NFM), or anti-rabbit (1:10000 in TBST) secondary antibodies for 1 h at RT. After additional three washes in TBST, proteins were visualized using Ez-ECL Kit (Biological industries, 20-500-120).

### Quantification

The labeled protein bands in immunoblots were detected using a gel documentation apparatus (XRS; Bio-rad) and analyzed using the Quantity one (4.5.0.) software. Background was subtracted from measured band. The levels of ABL1, Bog, and Tau were calculated as the ratio between the signals from the proteins and the signal from the antibody directed against Tubulin. In order to enable a comparison between the three groups, we normalized the signals by dividing the protein (ABL1, bog, or Tau)/tubulin signal obtained above in each individual rat taken from the paired, unpaired or naïve groups by the average respective protein (ABL1, bog, or Tau)/tubulin value of the naïve group (the baseline value).

### Statistical analysis

Analysis of behavioral data between two groups was done by independent Student's *t*-test. In Western blot experiment significance between groups was assessed by one-way ANOVA for the three groups (paired, unpaired, and naïve) followed by *post-hoc* LSD test.

## Results

### Paired CS-US training leads to fear conditioning whereas unpaired presentation of CS and US to safety learning

**Figure 1:**
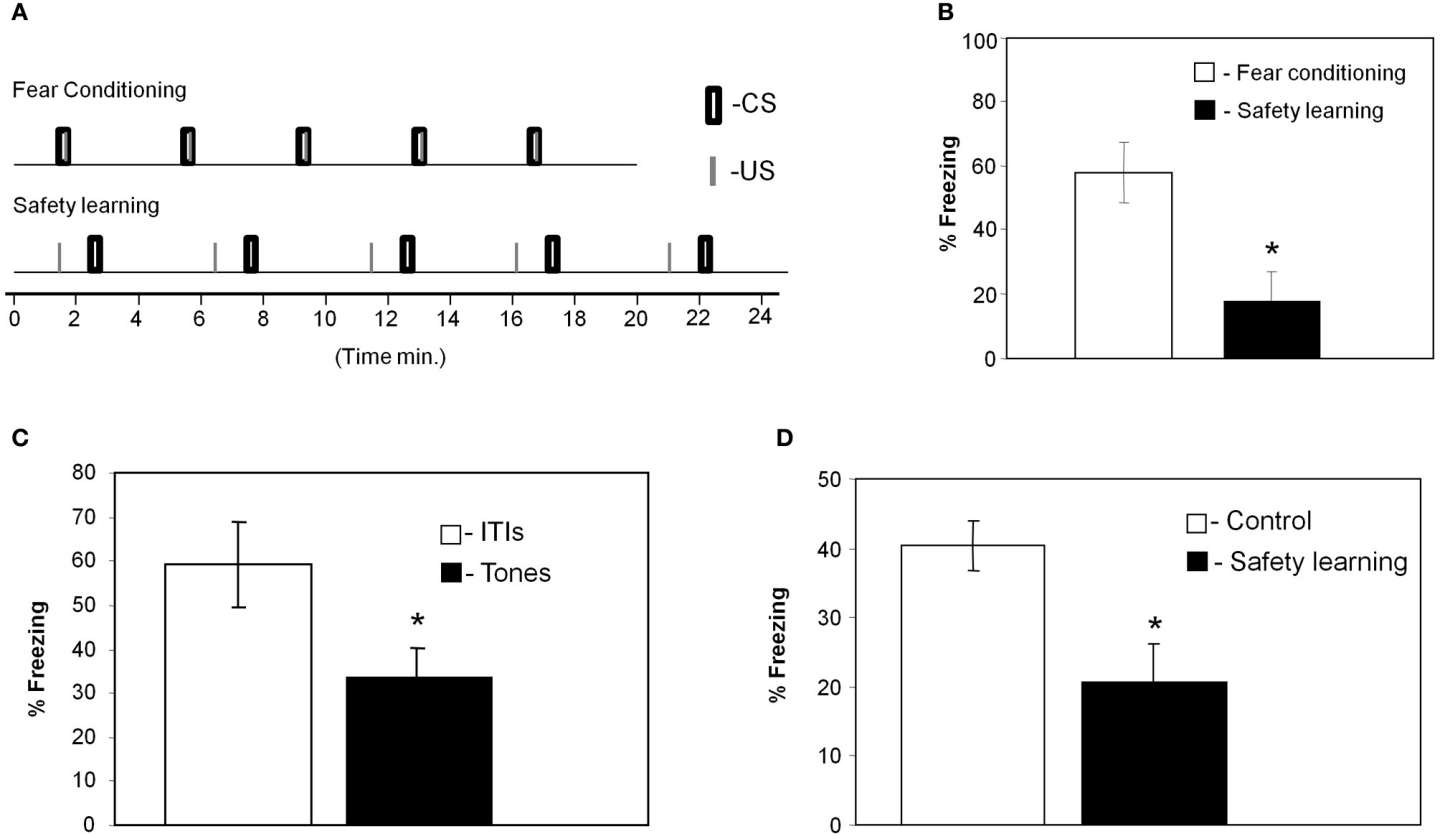
**Paired presentation of tone and shock leads to fear conditioning whereas unpaired presentation to safety learning. (A)** Training protocols for fear conditioning and safety learning indicating the exact timing of tones and footshocks presentations. **(B)** Rats that received paired presentation of tone and shock froze significantly more during test tones when compared to animals that received unpaired presentation (^*^p < 0.01) when tested 24 h after training indicating that they were fear conditioned to the tone CS. **(C)** In summation test animals that received tone and shock presentation in an unpaired manner froze significantly more during the ITIs than during the tone presentations when tested 24 h later in the same context where they were trained (^*^p < 0.05; *n* = 10). This result indicates that the animals that received unpaired presentation of tone and footshock learned that the CS is safe and predicts the absent of the shock. **(D)** In the retardation test animals were first trained with unpaired CS and US whereas control rats were not. The next day both groups were trained with fear conditioning protocol. Animals were tested a day later for freezing during tones presentation. Unpaired rats froze less than controls (^*^p < 0.03) showing that the tone acquires safety properties and excites fear less readily (*n* = 11 for safety group, *n* = 12 for control group).

We trained the animals with the fear conditioning or safety learning protocols (Figure 1A) and tested their response to the CS 24 h after training (these groups were trained together with the animals that were sacrificed for the microarray experiments). As shown in Figure 1B animals that were trained with paired stimulation (*n* = 8) froze significantly more (*p* < 0.01) during the tone when compared to the unpaired trained rat group (*n* = 7) showing that paired training leads to fear memory of the tone. We then used two standard tests, summation and retardation of acquisition, to establish that the unpaired trained animals memorized the tone as a safety signal. The summation test demonstrates that the tone can suppress freezing induced by the context which serves as the fearful stimulus (Rescorla, 1971; Williams et al., 1992). In summation test (Rescorla, 1971), animals were given safety training (unpairing of tone and shock) and 1 day later were returned to the shock context and freezing was assessed during ITIs and tone presentations. The animals showed significant suppression of context freezing during the tones when compared to their freezing during ITI (*p* < 0.05; Figure 1C). To rule out attentional or excitatory effects of the tone CS, we performed the retardation test (Rescorla, 1971). In the retardation paradigm rats were first trained for safety learning (unpaired presentation of US and CS) whereas control animals were not. Both groups were trained a day later for fear conditioning. When tested the next day, rats that were trained for safety learning showed less freezing to the tone than did control rats (*p* < 0.03; Figure 1D). Thus, in safety learning the tone acquires safety properties and excites fear less readily when subsequently paired with shock.

### Proteins levels in thalamus differ between fear conditioning and safety learning trained rats

**Figure 2:**
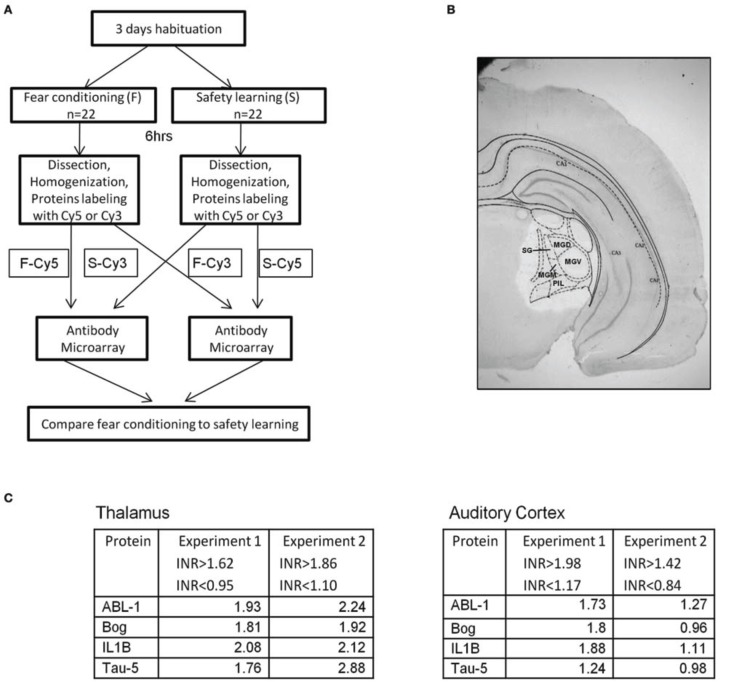
**Proteins levels in thalamus are lower in unpaired compared to paired trained rats. (A)** Rats were trained for fear conditioning [*F*; paired CS-US presentation (*n* = 22)] or safety learning [S; unpaired CS-US presentation (*n* = 22)]. Six hours after training the thalamus area, that includes the auditory and noniceptive areas was dissected, proteins were extracted, labeled, and subjected to antibody microarray. Differences between the level of specific proteins in the fear conditioning and safety learning groups were evaluated. **(B)** Representative brain section showing dissection of thalamus. **(C)** The levels of ABL1, Bog, IL1B, and Tau proteins in thalamus were found to be lower in the group trained for safety learning compared to the fear conditioning group 6 h after training. INR cut off indicates the upper and lower INR levels in which protein differences between fear conditioning and safety learning are taken into consideration. The levels of these proteins were not altered in auditory cortex. MGD, medial geniculate nucleus-dorsal; MGM, medial geniculate nucleus-medial; MGV, medial geniculate nucleus-ventral; PIL, post intralaminar thalamic nucleus; SG, Suprageniculate thalamic nucleus.

We were interested to screen changes in the level of proteins in auditory thalamus following fear conditioning or safety learning training. Toward that end we dissected thalamic areas that receive auditory and shock stimuli (e.g., Bordi and LeDoux, 1994) 6 h after training (Figures 2A,B). Molecular changes (e.g., gene expression) occur after fear conditioning around this time point (Lamprecht et al., 2009). The proteins were extracted and subjected to antibody microarray for analysis. Proteins level was compared between fear conditioning (*n* = 22) and safety learning (*n* = 22) trained rats (*n* = 11 pooled in each group in two separate experiments). Analysis of microarray revealed reproducibility between duplicate spots containing the same antibody. ABL1, Bog, IL1B, and Tau proteins were the only proteins that their level was changed in all experiments. The INR of proteins was above the cutoff indicating that their level in the unpaired group is lower compared to the paired group (Figure 2C). The level of these proteins was not different in these animals in the auditory cortex (Figure 2C).

### ABL1 level in auditory thalamus is reduced following safety learning

**Figure 3:**
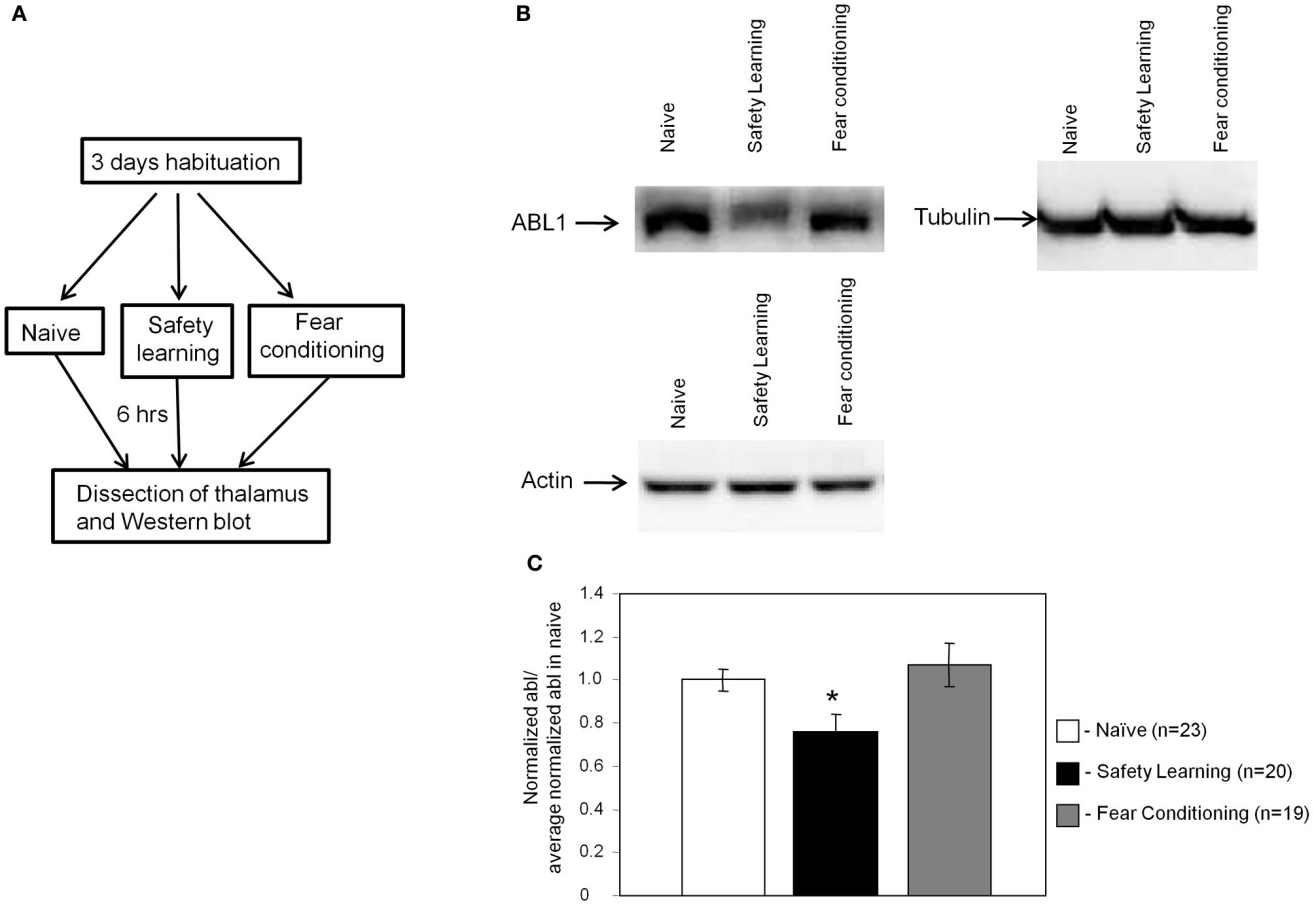
**ABL1 level in auditory thalamus is reduced following safety learning. (A)** Animals were trained for fear conditioning (*n* = 19), safety learning (*n* = 20) or left naïve [exposed to conditioning chamber only (*n* = 23)]. Six hours after training the thalamus was dissected, proteins were extracted and subjected to Western blot analysis with the anti-ABL1 or the control anti-tubulin or anti-actin as primary antibodies. **(B)** Representative Western blots of ABL1, tubulin and actin in various animal groups. **(C)** The ratio of normalized ABL1 of various experimental groups to the averaged normalized ABL1 in naïve is shown. ABL1 protein level is lower in safety learning trained group [*F*_(2)_= 4.195, ^*^p < 0.03] when compared to the fear conditioning (*p* < 0.009) or naïve (*p* < 0.04) groups. The level of ABL1 in fear conditioning group was not different from its level in the naïve group (*p* = 0.507).

We performed an experiment to monitor the level of Bog, Tau, and ABL1 in thalamus using the Western blot technique. The aims of this experiment were twofold. First, to verify the results detected in the microarray experiment and second to reveal whether the level of these proteins is reduced following safety learning or increased after fear conditioning. Toward that end we introduced an additional naïve group to monitor the basal level of the proteins in thalamus. The level of ABL1, Bog, and Tau was normalized to tubulin. Rats were scarified 6 h after fear conditioning, safety learning or naive training and brain area that includes the auditory thalamus was dissected (as in Figure 2B). Protein homogenate of each rat was monitored to detect the levels of ABL1, Bog, and Tau. As shown in Figure 3 the level of ABL1 protein was significantly reduced in safety learning group (*n* = 20) [*F*_(2)_= 4.195, *p* < 0.03] when compared with fear conditioning (*p* < 0.009; *n* = 19) or naïve group (*p* < 0.04; *n* = 23). The level of ABL1 in paired group was not different from its level in the naïve group (*p* = 0.507). The level of Bog and Tau showed similar trend in differences in protein levels as these observed in the microarray experiment [lower in unpaired (Tau = 0.9 ± 0.08; Bog = 0.86 ± 0.07) compared to naïve (Tau = 1 ± 0.06; Bog = 1 ± 0.07) and paired (Tau = 0.96 ± 0.08; Bog = 0.98 ± 0.08); *n* = 21; *n* = 23; *n* = 19 respectively] but these differences are smaller than these observed with ABL and are not significant [Tau = *F*_(2)_= 0.39, *p* = 0.6; Bog = *F*_(2)_= 1, *p* = 0.37]. The disparity in the Bog and Tau results between the microarray and Western blot studies could plausibly derive from the different methods used in the collection of the tissue and protein measurements. In the microarray experiments tissues from all animals were pooled to achieve the protein concentration needed, whereas in the Western blot the protein level was measured in individual rats: the latter methodology is necessary for quantification but it may introduce noise. We performed an additional experiment to explore whether the changes in ABL1 protein level occur in additional thalamic nuclei. We dissected a thalamic area more frontal and medial than the auditory thalamus that includes the ventral parts of the thalamus at the area of the ventrolateral and ventromedial thalamic nuclei and ventral posteromedial and posterolateral thalamic nuclei. There are no significant changes in the level of ABL1 between the paired, unpaired and naïve groups [*F*_(2)_= 0.006, *p* = 0.99; *n* = 4 each]. Taken together the aforementioned results show that safety learning, but not fear conditioning, lead to reduction in the level of ABL1 in thalamus and that the thalamus is differentially activated by safety learning.

## Discussion

Paired tone-shock presentation leads to fear memory of the tone (Fanselow and LeDoux, 1999; LeDoux, 2000; Davis and Whalen, 2001; Sah et al., 2003; Maren, 2005) whereas unpaired training leads to safety learning where the rats remember the CS as a safety signal that predicts the absent of the shock (Rogan et al., 2005; Pollak et al., 2008; Ostroff et al., 2010). In this study we find that safety learning, but not fear conditioning, leads to reduction of ABL1 protein level in a thalamic region that includes areas that process the tone and shock information. Furthermore, ABL1 level was not different in auditory cortex between safety or fear learning. Thus, safety learning differentially activates the thalamus leading to modulation of ABL1 protein level in this brain area.

Studies have shown that the auditory thalamus is needed for safety learning. For example, rats were given feature-negative discrimination training in which a noise was conditioned to inhibit fear to a light that signals danger. Following training, rats were given lesions at the auditory thalamus and after recovery were tested for fear inhibition in the presence of the noise safety signal. Lesions of auditory thalamus impaired the ability of the noise inhibitor to inhibit fear indicating the need of the thalamus for detecting the safety properties of the auditory stimulus (Heldt and Falls, 2006).

The auditory thalamus transfers the auditory information to the amygdala. Safety learning in mice induces long-lasting depression of CS-evoked activity in the LA, consistent with fear reduction (Rogan et al., 2005) whereas fear conditioning induces increase in CS-evoked responses in LA (Quirk et al., 1995; McKernan and Shinnick-Gallagher, 1997; Repa et al., 2004). Another study reported a decrease in amygdala response to an auditory CS− (unpaired with US) after discriminative CS+ (paired with US) training in the cat (Collins and Paré, 2000). The safety signal-driven inhibition of amygdala could mediate a shutdown of some aspects of amygdala function during the safety CS. It was shown that direct thalamic projection from MGm/PIN mediates the decreased CS-evoked activity in the LA after safety learning (Rogan et al., 2005).

What could be the implications of reduction in ABL1 protein following safety learning on cellular processes mediating memory formation? Abl is a tyrosine kinase that affects key neuronal function by regulating downstream effectors such as cytoskeletal proteins (Lanier and Gertler, 2000). Abl is localized in both the presynaptic terminals and dendritic spines in the hippocampus (Moresco et al., 2003). Within the presynaptic terminal, Abl localization is restricted to the active zone. In spines, Abl localization is prominent at the PSD. It was shown that chemical or genetic inhibition of c-Abl kinase activity reduces PSD-95 tyrosine phosphorylation, leading to reduced PSD-95 clustering and synapse number in treated cultured hippocampal neurons (de Arce et al., 2010). In addition, inhibition of c-abl activity reduced GluR1 cluster density in neurons (Lanier and Gertler, 2000). Furthermore, abl may have an effect on dendritic structure. Inhibition of Abl kinases in hippocampal culture leads to simplification of dendritic branching (Jones et al., 2004) and significant reduction in neurite branching and cortical neurons (Woodring et al., 2002). Such altered functions following reduction in abl activity may have a direct influence on synaptic efficacy in neurons. Abl protein may affect also presynapse functions. In abl^−/−^ mice Paired-pulse facilitation (PPF), a transient form of presynaptic plasticity, is reduced in hippocampal slices suggesting that abl is required for optimal neurotransmitter release (Moresco et al., 2003). Basal synaptic transmission, posttetanic potentiation (PTP), long-term potentiation (LTP), and long-term depression (LTD) were similar between wild-type and abl^−/−^ mice and in STI571-treated wild-type slices. These results indicate an important function of Abl in synaptic efficacy via a presynaptic mechanism during repetitive activation. Thus, since ABL1 may be involved in alterations in synaptic efficacy reduced ABL1 level after safety learning could contribute to changes in synaptic responses to the tone in thalamus and as a consequence alterations in activation of LA by the thalamus (Rogan et al., 2005).

What could be the mechanisms of rapid ABL1 protein level reduction observed after safety learning? Three mechanisms are suggested: (1) Ubiquitination dependent degradation: it was shown that activated c-abl is degraded by the ubiquitin-dependent proteasome pathway (Echarri and Pendergast, 2001); (2) Rapid increase in microRNA leading to reduction in ABL1 RNA and protein levels. Indeed, microRNA that reduces ABL1 levels, miR-203, was detected (Bueno et al., 2008). Moreover, microRNA can be induced rapidly after stimulation leading to synaptic plasticity (Park and Tang, 2009); (3) Repression of ABL1 gene expression.

The study shows that training leading to safety learning induces specific molecular changes different from fear conditioning in thalamic areas processing the tone and shock stimuli. Although both fear conditioning and safety learning utilize the same stimuli they may use different cellular mechanisms to form long-term memory. This observation is consistent with studies showing differential activation of gene expression (Pollak et al., 2008) and formation of spine morphology (Ostroff et al., 2010) in LA following safety and fear learning. Thus, memory formation of different sort may not engage identical cellular mechanism even if formed following the same sensory stimuli and in the same area. The temporal differences in presentation of the stimuli during safety or fear learning influence molecular activation following learning and may affect neurons differently to form distinct memories.

### Conflict of interest statement

The authors declare that the research was conducted in the absence of any commercial or financial relationships that could be construed as a potential conflict of interest.
